# Recent Trends and Advances in Telecommunications and Sensing

**DOI:** 10.3390/s24154772

**Published:** 2024-07-23

**Authors:** Erich Leitgeb, Maja Matijašević, Wilfried Gappmair, Mario Kušek

**Affiliations:** 1 Institute of Microwave and Photonic Engineering, Graz University of Technology, Inffeldgasse 12, 8010 Graz, Austria; erich.leitgeb@tugraz.at; 2 Faculty of Electrical Engineering and Computing, Telecommunications Department, University of Zagreb, Unska ul. 3, 10000 Zagreb, Croatia; maja.matijasevic@fer.hr (M.M.); mario.kusek@fer.hr (M.K.); 3 Institute of Communication Networks and Satellite Communications, Graz University of Technology, Inffeldgasse 12, 8010 Graz, Austria

In the context of the 17th International Conference on Telecommunications (ConTEL), which took place at Graz University of Technology, Austria, from the 11th until the 13th of July 2023, the chairs of the conference were approached by MDPI to organize a Special Issue as part of the *Sensors* journal (ISSN: 1424-8220). The authors of accepted and presented papers were invited to submit a modified or adapted version to MDPI. The Special Issue was launched and the MDPI office offered a 10% discount to authors of published papers. The name of the Special Issue was defined as “Recent Trends and Advances in Telecommunications and Sensing”, which seemed to be best suited for the contributions presented and published at ConTEL 2023.

The 17th International Conference on Telecommunications was hosted by the Faculty of Electrical Engineering at Graz University of Technology, Austria. Numerous sessions about photonic and optical communications, information and communication technologies, protocols and networks, multimedia applications, RF engineering and antennas were organized and chaired by experts in their fields. The program also included internationally renowned keynote and invited speakers presenting an overview of their current research activities. Of the 53 manuscripts submitted to the conference by authors from about 28 countries, exactly 37 papers were accepted. Each paper was evaluated by at least three independent reviewers, focusing on relevance and timeliness, technical content and scientific rigor, novelty and originality, as well as the quality of presentation. All accepted and registered contributions were published via IEEE Xplore.

All the authors were invited to work on an extended version of their conference paper and submit it to the Special Issue. However, manuscripts from authors not related to ConTEL 2023 were also considered. MDPI allowed submissions with a similar title, but around 50% or more of the content should be different to the content of the original paper. New and original contributions were also highly encouraged.

**Guest Editors**

- *Erich Leitgeb*: Graz University of Technology, Austria; Institute of Microwave and Photonic Engineering
- *Maja Matijašević*: University of Zagreb, Croatia; Faculty of Electrical Engineering and Computing, Department of Telecommunications
- *Wilfried Gappmair*: Graz University of Technology, Austria; Institute of Communication Networks and Satellite Communications
- *Mario Kušek*: University of Zagreb, Croatia; Faculty of Electrical Engineering and Computing, Department of Telecommunications

**Background**: With new services and access schemes, especially related to 5G and beyond, there is a growing need for improved terrestrial and non-terrestrial networking infrastructure, not just in terms of quality and performance, but also with respect to scalability, mobility, energy, spectral efficiency and integration of multiple technologies. Extended ConTEL 2023 contributions and original research papers were solicited. Analytical and theoretical investigations as well as experimental and practical work were also accepted.

**Topics** included, but not limited to the following areas:

- Optical wireless communications;
- Satellite and space communications;
- Antennas and wave propagation, including NFC and RFID;
- Internet of Things (IoT) and sensor networks;
- Smart spaces: context and situation-awareness;
- Internet and next-generation networking;
- Energy-efficient protocols and networks;
- Software-defined networks and network function virtualization;
- Multimedia applications, rich communication services and networked games;
- QoE and QoS assessment and provisioning;
- Machine learning and big data in telecommunications;
- Social networking and social media;
- Security and privacy issues in ICT services and networks;
- Digital inclusion and assistive technology in ICT services.

All in all, the following nine papers were accepted for publication in the Special Issue on “Recent Trends and Advances in Telecommunications and Sensing”:

1. **Dragana Krstic**, **Suad Suljovic**, **Goran Djordjevic**, **Nenad Petrovic** and **Dejan Milic**, “MDE and LLM Synergy for Network Experimentation: Case Analysis of Wireless System Performance in Beaulieu-Xie Fading and κ-µ Co-Channel Interference Environment with Diversity Combining”, *Sensors* 2024, *24* (10), 3037; May 2024.
2. **Eleni Niarchou**, **Vicente Matus**, **Jose Rabadan**, **Victor Guerra** and **Rafael Perez-Jimenez**, “Optical Camera Communications in Healthcare: A Wearable LED Transmitter Evaluation during Indoor Physical Exercise”, *Sensors* 2024, *24*(9), 2766; April 2024.
3. **Slawomir Hanczewski**, **Maciej Stasiak** and **Michal Weissenberg**, “An Analytical Model of IaaS Architecture for Determining Resource Utilization”, *Sensors* 2024, *24*(9), 2758; Apr. 2024.
4. **Adrian Komadina**, **Ivan Kovačević**, **Bruno Štengl** and **Stjepan Groš**, “Comparative Analysis of Anomaly Detection Approaches in Firewall Logs: Integrating Light-Weight Synthesis of Security Logs and Artificially Generated Attack Detection”, *Sensors* 2024, *24*(8), 2636; Apr. 2024.
5. **Jay Bojič Burgos** and **Matevž Pustišek**, “Decentralized IoT Data Authentication with Signature Aggregation”, *Sensors* 2024, *24*(3), 1037; Feb. 2024.
6. **Lenka Benova** and **Ladislav Hudec**, “Comprehensive Analysis and Evaluation of Anomalous User Activity in Web Server Logs”, *Sensors* 2024, *24*(3), 746; Jan. 2024.
7. **Mariusz Głąbowski**, **Maciej Sobieraj** and **Maciej Stasiak**, “Analytical Model of the Connection Handoff in 5G Mobile Networks with Call Admission Control Mechanisms”, *Sensors* 2024, *24*(2), 697; Jan. 2024.
8. **Wilfried Gappmair**, “Novel Results on SNR Estimation for Bandlimited Optical Intensity Channels”, *Sensors* 2024, *24*(1), 23; Dec. 2023.
9. **Khawaja Tahir Mehmood**, **Shahid Atiq** and **Muhammad Majid Hussain**, “Enhancing QoS of Telecom Networks through Server Load Management in Software-Defined Networking (SDN)”, *Sensors* 2023, *23*(23), 9324; Nov. 2023.

